# Characteristics of Intestinal Microbiota in Japanese Patients with Mild Cognitive Impairment and a Risk-Estimating Method for the Disorder

**DOI:** 10.3390/biomedicines11071789

**Published:** 2023-06-22

**Authors:** Kouta Hatayama, Aya Ebara, Kana Okuma, Hidetaka Tokuno, Kazumi Hasuko, Hiroaki Masuyama, Iyoko Ashikari, Takuji Shirasawa

**Affiliations:** 1 Symbiosis Solutions Inc., Tokyo 101-0064, Japan; 2 Ashikari Clinic, Tokyo 164-0011, Japan; 3 Ochanomizu Health and Longevity Clinic, Tokyo 101-0062, Japan

**Keywords:** intestinal microbiota, mild cognitive impairment, MCI, structural equation modeling, risk estimation

## Abstract

Intestinal microbiota may play a significant role in the development and progression of mild cognitive impairment (MCI). In addition, sex differences in the prevalence of MCI and intestinal microbiota are likely to exist. Therefore, this study investigated the association between MCI and intestinal microbiota by comparing Japanese patients in their 70s with MCI (11 males and 18 females) and disease-free controls (17 males and 23 females), taking sex into account. In both sexes, *Clostridium*_XVIII, *Eggerthella*, *Erysipelatoclostridium*, *Flavonifractor*, and *Ruminococcus* 2 were the more abundant taxa in the MCI group, whereas *Megasphaera*, *Oscillibacter*, *Prevotella*, *Roseburia*, and *Victivallis* were less abundant. Based on these characteristics, it was hypothesized that the composition of the intestinal microbiota in the MCI group leads to dysregulation of the intestinal microbiota, increased intestinal and blood–brain barrier permeability, and increased chronic neuroinflammation, with the long-term persistence of these abnormalities ultimately leading to cognitive decline. Furthermore, risk estimation models for MCI based on intestinal microbiota data were developed using structural equation modeling. These tests discriminated between the MCI and control groups. Incorporating these factors into intestinal microbiota testing using stool samples may be an efficient method to screen individuals with MCI.

## 1. Introduction

Mild cognitive impairment (MCI) is a cognitive condition that is intermediate between normal and dementia, but is not dementia [1]. The prevalence of MCI is higher in the elderly, increases with lower educational levels, and tends to be higher in males [2,3]. In patients with MCI, cognitive decline is present but does not significantly interfere with daily life. However, patients with MCI are at high risk of progressing to dementia (e.g., Alzheimer’s disease, frontotemporal dementia, dementia with Lewy bodies, and Parkinson’s dementia) [2,4]. Recovery is difficult once dementia develops. However, MCI may return to normal cognitive function, and appropriate interventions for patients with MCI may improve their cognitive function or prevent progression to dementia [2,3]. To achieve this, it is important to identify individuals with MCI or those at a high risk of MCI at an early stage. Understanding the mechanisms of onset and progression is essential for effective treatment and prevention.

Cognitive function may be affected by intestinal microbiota. An unfavorable composition of intestinal microbiota (i.e., dysbiosis) can lead to increased intestinal barrier permeability, systemic inflammation, impairment of the blood–brain barrier (BBB), promotion of neuroinflammation, and ultimately, neurodegeneration [5,6]. However, its exact mechanism of action has not yet been elucidated. Several studies have suggested an association between MCI and intestinal microbiota [7,8,9,10,11,12,13]. Cognitive decline in MCI may also be affected by intestinal microbiota, but further studies are needed to understand this.

Previously, Hatayama et al., reported sex differences in the intestinal microbiota of a Japanese population [14]. The study suggests that there may be sex-related differences in disease-associated intestinal bacteria. In addition, sex is included as a risk factor for MCI [2,3]; therefore, studies on the association between MCI and intestinal microbiota should incorporate a sex-difference perspective. However, most previous studies on the association between MCI and intestinal microbiota included mixed populations of males and females in their analyses and did not consider sex differences.

Therefore, this study investigated the association between MCI and intestinal microbiota by comparing Japanese patients in their 70s with MCI and disease-free controls, taking sex differences into account. Specifically, in addition to the analysis of mixed-sex groups, a reanalysis of the groups by sex was performed to understand the association between MCI and intestinal microbiota. Consequently, it is possible to hypothetically explain some of the mechanisms underlying MCI associated with intestinal bacteria. In addition, this study developed models for estimating the risk of MCI in males and females, using the identified intestinal bacteria associated with MCI as observed variables. Incorporating these MCI risk estimation models into intestinal microbiota testing using stool samples could be an effective screening method for individuals with MCI or those at high risk of MCI.

## 2. Materials and Methods

### 2.1. Study Participants

Twenty-nine Japanese individuals in their 70s (11 males and 18 females) diagnosed with MCI at medical institutions in Japan (Ochanomizu Health and Longevity Clinic, Tokyo, Japan, or Ashikari Clinic, Tokyo, Japan) were included in the MCI group (Table 1). The patients with MCI also had other diseases (Supplementary Table S1). One male and one female in the MCI group had used antibiotics within the past three months. Stool samples were collected from the MCI group between August 2021 and June 2022.

Forty disease-free Japanese individuals in their 70s (17 males and 23 females) were selected from users of the intestinal microbiota testing service provided by Symbiosis Solutions, Inc. (Tokyo, Japan) based on a questionnaire, and served as the control group (Table 1). None of the males or females in the control group had used antibiotics in the past three months. Stool samples from the control group were collected between May 2020 and July 2022.

Those with insufficient data on intestinal microbiota, non-Japanese ethnicity, or enema stools were excluded from the study.

### 2.2. Ethical Considerations

The study was conducted according to the guidelines of the Declaration of Helsinki and approved by the Institutional Review Board of Shiba Palace Clinic (Tokyo, Japan) (approval number and date: 144131_rn-27593, 9 January 2020; 145968_rn-29327, 12 November 2020). Written informed consent was obtained from all the participants.

### 2.3. Questionnaire Survey

Background information, including age, sex, and health status (including disease status other than MCI), was collected from the participants’ self-reports using a questionnaire.

### 2.4. Stool Samples

Stool samples were collected by the participants themselves using a stool collection kit (Techno-Suruga Laboratory Co., Ltd., Shizuoka, Japan) [15]. The stool samples were then mailed to the laboratory without temperature control.

### 2.5. 16S rRNA Data Analysis

DNA extraction from stool samples and sequencing using the MiSeq system (Illumina, San Diego, CA, USA) were performed following the methods described by Kono et al. [15]. The sequence of the 16S rRNA gene (variable regions V1-V3) was determined using the 35F and 520R primers. Data analysis of the 16S rRNA gene sequences was performed as described by Hatayama et al. [14]. Amplicon Sequence Variants (ASVs) were created using the DADA2 v. 1.16.0 package [16] in R software v. 4.0.3 (R Foundation for Statistical Computing, Vienna, Austria) [17]. The taxonomic affiliation of ASVs was determined using the Ribosomal Database Project (RDP) training set v. 18 [18] (available online at https://zenodo.org/record/4310151#.ZDUBAXbP2Ht; accessed on 11 April 2023).

### 2.6. Analysis of Intestinal Microbiota

The α-diversity indices (Shannon, Simpson, and Pielou) were calculated at the genus level using the vegan package v. 2.6-2 in R v. 4.2.0. The number of taxa at the genus level was counted as richness.

To visualize β-diversity, non-metric multidimensional scaling (NMDS) based on the Bray–Curtis index was used. The metaMDS function in R v. 4.2.0 vegan v. 2.6-2 package was used for NMDS. Permutational Multivariate Analysis of Variance (PERMANOVA) was performed using the vegan adonis function v. 2.6-2, with 9999 permutations.

Comparison of the intestinal microbiota between the two groups was performed at the genus level using the R v. 4.2.0 package ALDEx2 v. 1.28.1 [19], according to the methods by Kono et al. [15]. For this comparison, microbiota abundance count data were transformed into a centered log ratio (CLR).

### 2.7. Statistical Analysis of Data Excluding Intestinal Microbiota Data

The Wilcoxon rank-sum test was used to compare the data between groups. The test was performed using the Wilcoxon test function (paired = FALSE, correct = FALSE) in R, v. 4.2.0. The chi-square test was performed using the chi-square test function in R v. 4.2.0. Statistical significance was set at *p* < 0.05.

### 2.8. MCI Risk Estimation Modeling

To estimate the risk of disease from intestinal microbiota using structural equation modeling (SEM), we followed the method reported by Tokuno et al. [20] by constructing MCI risk estimation models using R v.4.2.2. The intestinal microbiota data were CLR-transformed using the aldex.clr function in the R v. 4.1.0 package ALDEx2 v. 1.24.0.

SEMs were constructed using CLR-transformed intestinal microbiota data from the MCI and control groups and their MCI morbidity data. The SEMs were performed using the cfa function in the lavaan v 0.6-12 package [21] with the arguments std.lv = TRUE, std.ov = TRUE, ordered = “disease_flg”, and check.gradient = TRUE. The cfa function was estimated using the diagonally weighted least-squares method. The latent variable values in the SEMs were obtained using the lavPredict function in lavaan using the Empirical Bayes Method (EBM). By extracting the measurement equation part of the SEMs, we constructed new SEMs that were used to calculate the latent variable values in the MCI morbidity data-blinded situation. The latent variable values from the new SEMs were obtained using the lavPredict function with the EBM. These latent variable values from the new SEMs were used as explanatory variables in the construction of the MCI risk estimation model.

The MCI risk estimation models were constructed using logistic regression analysis. Model training and 10-fold cross-validations were performed with the trainControl and train functions in the caret v 6.0-93 package [22]. We set the arguments for the trainControl: method = “cv”, number = 10, summaryFunction = caret::twoClassSummary, classProbs = TRUE, savePredictions = TRUE, sampling = “smote”, and the arguments for the train: method = “glm”, family = “binomial”, trControl = cv, metric = “ROC”. The themis package (v. 1.0.0) [23] was used for SMOTE sampling in the training function. The roc and ggroc functions in the pROC v. 1.18.0 package [24] were used for receiver operating characteristic (ROC) analyses and to draw the ROC curves.

## 3. Results

### 3.1. Comparison of Intestinal Microbiota between MCI and Control Groups (Mixed Sex)

The MCI and control groups comprised 29 Japanese individuals diagnosed with MCI and 40 disease-free participants, respectively. The participants in the MCI and control groups were in their 70s; however, there was a significant difference in terms of age (Table 1). There were no significant differences in the sex composition or BMI between the two groups (Table 1).

**Figure 1:**
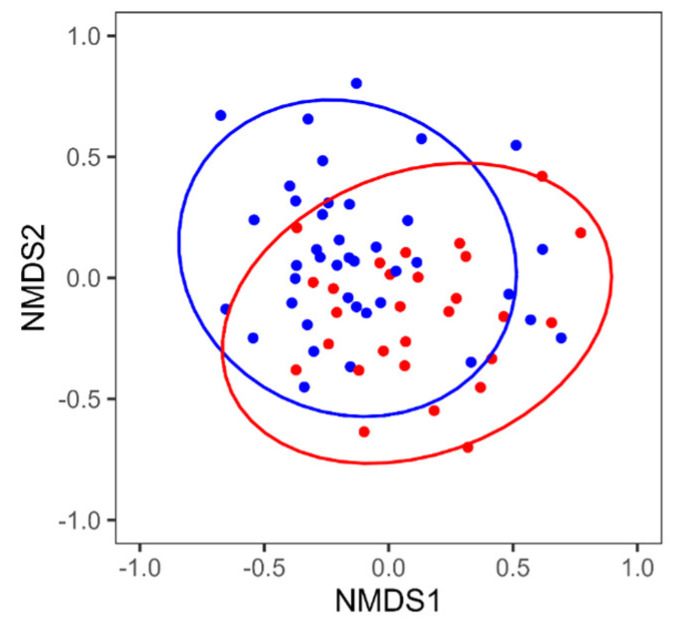
Non-metric multidimensional scaling (NMDS) plot using the Bray–Curtis index of the intestinal microbiota of the mixed-sex MCI and control groups (stress: 0.19, PERMANOVA *p*-value = 0.010). Red and blue dots in the NMDS plot indicate samples of MCI and control groups, respectively. Ellipses in the NMDS plot indicate 95% confidence intervals around the centroid.

The diversity of intestinal microbiota in the MCI and control groups was compared. The intestinal microbiota of each participant was determined by 16S rRNA gene (V1-V3 region) amplicon sequencing. Shannon, Simpson, Richness, and Pielou diversity indices were used for α-diversity analysis, all of which showed no significant differences between the MCI and control groups (Table 1). The β-diversity analysis by NMDS and PERMANOVA showed that the intestinal microbiota of the MCI and control groups differed in their composition (Figure 1).

**Figure 2:**
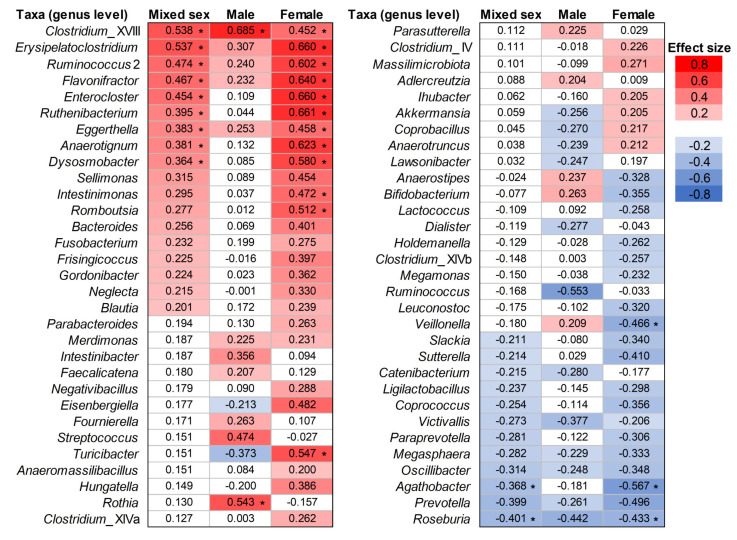
Intestinal bacterial taxa associated with MCI (genus level). Taxa with values of ALDEx2 effect size above 0.2 (more abundant taxa in the MCI group) and taxa with values below −0.2 (less abundant taxa in the MCI group) are shown as red and blue gradients according to their values, respectively. An asterisk indicates that the *p*-value obtained from the Wilcoxon rank-sum test is less than 0.05. All Benjamini–Hochberg corrected *p*-values were greater than 0.05.

To identify the intestinal bacteria that differed between the MCI and control groups, an analysis was performed using the ANOVA-Like Differential Expression tool (ALDEx2). Taxa (genus level) with ALDEx2 effect size values greater than 0.2 were defined as more abundant while taxa with values less than −0.2 were considered less abundant in the MCI group. Eighteen taxa (*Clostridium*_XVIII, *Erysipelatoclostridium*, *Ruminococcus* 2, *Flavonifractor*, *Enterocloster*, *Ruthenibacterium*, *Eggerthella*, *Anaerotignum*, *Dysosmobacter*, *Sellimonas*, *Intestinimonas*, *Romboutsia*, *Bacteroides*, *Fusobacterium*, *Frisingicoccus*, *Gordonibacter*, *Neglecta*, and *Blautia*) were observed to be more abundant in the MCI group, and 12 taxa (*Roseburia*, *Prevotella*, *Agathobacter*, *Oscillibacter*, *Megasphaera*, *Paraprevotella*, *Victivallis*, *Coprococcus*, *Ligilactobacillus*, *Catenibacterium*, *Sutterella*, and *Slackia*) were less abundant in the MCI group (Figure 2, Mixed sex).

### 3.2. Reanalysis of the MCI and Control Groups by Sex

Sex is considered a risk factor for MCI [2,3]. Furthermore, sex differences in the intestinal microbiota of Japanese individuals have been reported [14]. These findings suggest that the intestinal bacteria associated with MCI may differ by sex; however, it was difficult to observe sex-related results in the analysis of mixed-sex groups. Therefore, the MCI and control groups were subdivided according to sex, and their intestinal microbiota was reanalyzed.

There was a significant difference in age between the MCI and control groups for males (Table 2) but not for females (Table 3). There were no significant differences in BMI between the MCI and control groups for males or females (Table 2 and Table 3).

**Figure 3:**
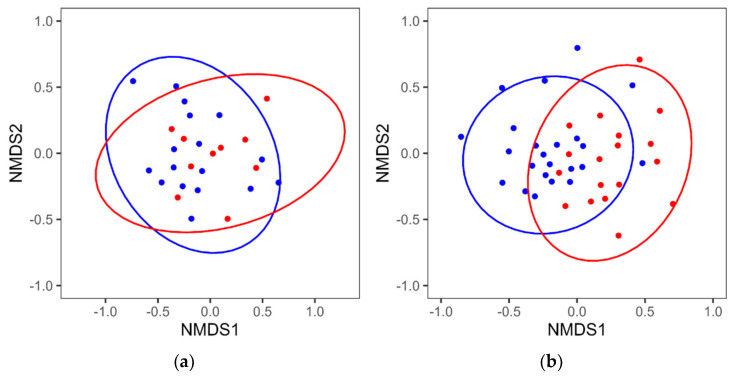
NMDS plot using the Bray–Curtis index of the intestinal microbiota of the MCI and control groups. (**a**) NMDS plots of males (stress = 0.15; PERMANOVA *p*-value = 0.216); (**b**) females (stress = 0.19; PERMANOVA *p*-value = 0.010). Red and blue dots in the NMDS plots indicate samples of MCI and control groups, respectively. Ellipses in the NMDS plots indicate 95% confidence intervals around the centroid.

Analyses by sex showed no significant differences in the four α-diversity indices, similar to the mixed-sex analyses (Table 2 and Table 3). In the β-diversity analysis, significant differences were found in the composition of the intestinal microbiota between the MCI and control groups in females (PERMANOVA *p*-value = 0.010) but not in males (*p*-value = 0.216) (Figure 3).

ALDEx2 analysis showed that 16 taxa were more abundant, and 14 taxa were less abundant in the male MCI group (Figure 2, Male). In the female MCI group, 32 and 19 taxa were observed as more and less abundant, respectively (Figure 2, Female). Eleven of the taxa associated with MCI observed in males were consistent with the results of mixed-sex MCI (more abundant taxa in the male and mixed-sex MCI groups: *Clostridium*_XVIII, *Erysipelatoclostridium*, *Ruminococcus* 2, *Flavonifractor* and *Eggerthella*; less abundant taxa in the male and mixed-sex MCI groups: *Roseburia*, *Prevotella*, *Oscillibacter*, *Megasphaera*, *Victivallis*, and *Catenibacterium*). In contrast, 29 of the taxa associated with MCI observed in females were consistent with the results of mixed sex (i.e., the taxa associated with MCI observed in mixed sex were consistent with those of females, except for *Catenibacterium*). This indicates that the taxa associated with MCI observed in the mixed-sex analysis were strongly influenced by females.

In the sex-specific reanalysis, some taxa were observed as intestinal bacteria associated with MCI, which were not observed in the mixed-sex analysis. In the male MCI group, 11 taxa (*Rothia*, *Streptococcus*, *Intestinibacter*, *Bifidobacterium*, *Fournierella*, *Anaerostipes*, *Merdimonas*, *Parasutterella*, *Veillonella*, *Faecalicatena*, and *Adlercreutzia*) were newly identified as more abundant, while eight taxa (*Ruminococcus*, *Turicibacter*, *Dialister*, *Coprobacillus*, *Akkermansia*, *Lawsonibacter*, *Anaerotruncus*, and *Eisenbergiella*) were newly identified as less abundant (Figure 2, Male). In the female MCI group, 14 taxa (*Turicibacter*, *Eisenbergiella*, *Hungatella*, *Negativibacillus*, *Massilimicrobiota*, *Parabacteroides*, *Clostridium*_XlVa, *Merdimonas*, *Clostridium*_IV, *Coprobacillus*, *Anaerotruncus*, *Akkermansia*, *Ihubacter*, and *Anaeromassilibacillus*) were newly observed as more abundant and eight taxa (*Veillonella*, *Bifidobacterium*, *Anaerostipes*, *Leuconostoc*, *Holdemanella*, *Lactococcus*, *Clostridium*_XlVb, and *Megamonas*) were newly observed as less abundant (Figure 2, Female). There were also taxa that showed conflicting associations with MCI based on sex (*Eisenbergiella*, *Turicibacter*, *Akkermansia*, *Coprobacillus*, *Anaerotruncus*, *Anaerostipes*, *Bifidobacterium* and *Veillonella*). These results indicate that there are differences in the taxa associated with MCI according to sex. Reanalysis considering sex provided information on the intestinal microbiota associated with sex differences that would have been missed if only a mixed-sex analysis was performed.

### 3.3. Risk Estimation for MCI Based on Intestinal Microbiota

**Figure 4:**
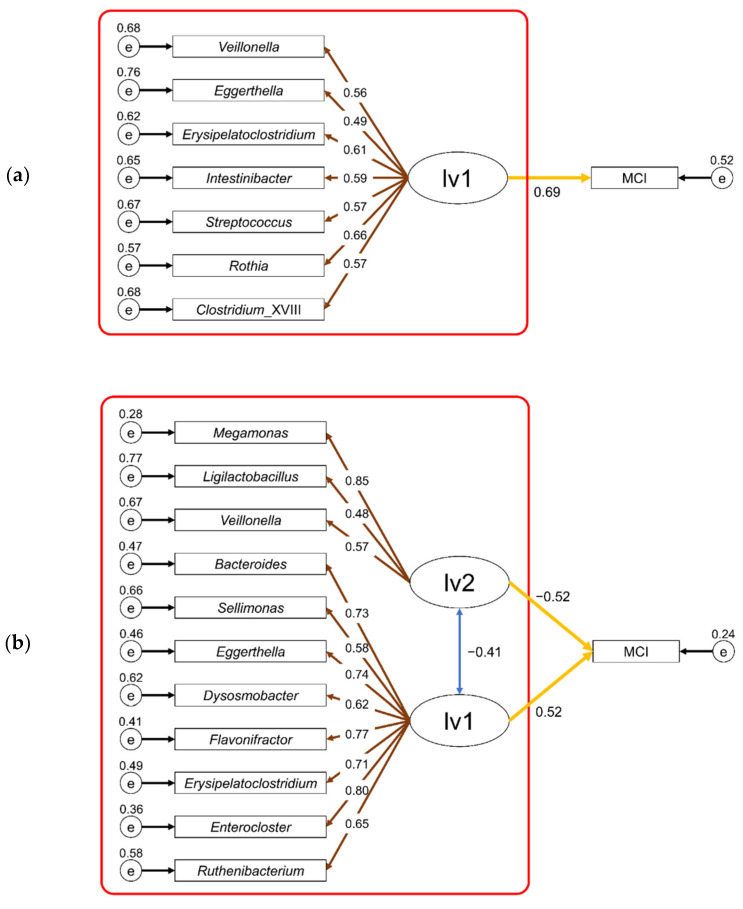
Constructed structural equation models for males (**a**) and females (**b**) and their values for each parameter. Ellipse (lv1 or lv2), rectangle (MCI or taxa name) and circle (e) represent the latent variable, observed variable, and residual terms, respectively. The values above the small circle, double-headed blue arrow, brown arrow, and yellow arrow represent residual variance, correlation coefficient, loading value, and path coefficient, respectively. The detailed results for males (**a**) and females (**b**) are shown in Supplementary Tables S2 and S3, respectively. The red box indicates the portion used to estimate latent variable values.

Tokuno et al. [20] reported a method using SEM to estimate the risk of disease based on the composition of the intestinal microbiota. In this study, we attempted to estimate the risk of MCI using this method. Based on the study by Tokuno et al. [20], we constructed an SEM composed of two latent variables using the CLR-transformed intestinal bacterial abundance as the observed variable. In the SEM for each sex, all more abundant taxa in the MCI group were assigned to latent variable 1 (lv1) and all less abundant taxa in the MCI group were assigned to latent variable 2 (lv2) as observed variables (indicators). Starting from the SEM for each sex, the observed variables were reduced until an SEM was established that had goodness-of-fit index (GFI) and adjusted GFI (AGFI) values close to 1 and a root mean square error of approximation (RMSEA) close to 0. In the final SEM constructed for females (GFI = 0.94, AGFI = 0.89, RMSEA ≤ 0.01), eight observed variables (taxa) were assigned to lv1, and three to lv2 (Figure 4b). In contrast, the final SEM constructed for males, in which seven observed variables were assigned to lv1 and two to lv2, showed a strong correlation between these two latent variables (Supplementary Figure S1). High correlations between latent variables were undesirable because they led to multicollinearity in the MCI risk estimation model. In addition, the high correlation between lv1 and lv2 suggests that either latent variable alone could explain MCI. Therefore, the next attempt was to construct a male SEM consisting only of lv1. For lv1, we selected all the taxa that were more abundant in the male MCI group and assigned them as observed variables. Using the SEM as the starting point, the observed variables were reduced using the method described above. An SEM (GFI = 0.96, AGFI = 0.91, RMSEA ≤ 0.01) was constructed for males with seven observed variables assigned to lv1 (Figure 4a).

For each sex, we extracted the measurement equation portion of the initial SEM in Figure 4 (red box) and constructed a new SEM to estimate the latent variable values (males: lv1; females: lv1, lv2) when the participants’ MCI morbidity was unknown. The estimated latent variable values for each participant were calculated using these SEMs. Finally, a logistic regression model was used to construct a risk estimation model for MCI by sex, with the estimated latent variable values as explanatory variables.

**Figure 5:**
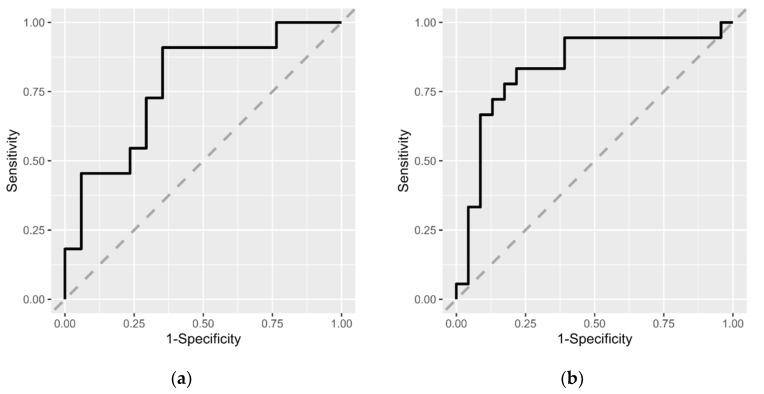
Receiver operating characteristic (ROC) curves for the risk estimation models of MCI for males (**a**) and females (**b**). Area under the curves (AUCs) for the risk estimation models of MCI for males and females were 0.75 and 0.87, respectively. Gray dashed line represents the ROC curve for a random estimate.

For the risk estimation models of MCI for each sex, we tested the predictive accuracy of each model using ROC curve analysis (Figure 5). The results showed that the area under the curves (AUCs) for the risk estimation models of MCI for males and females were 0.75 and 0.87, respectively. This indicates that risk estimation models for MCI based on intestinal microbiota data are effective in predicting MCI morbidity status.

## 4. Discussion

We investigated the intestinal microbiota associated with MCI by comparing the intestinal microbiota of Japanese patients with MCI with that of a disease-free control group. Most previous studies on the association between MCI and intestinal microbiota have been analyzed in mixed-sex groups [7,8,9,10,11,12,13]. In contrast, this study was based on the fact that sex is one of the risk factors for MCI [2,3] and the existence of sex differences in the intestinal microbiota [14]; therefore, the analysis was not only performed on mixed-sex groups but the data were also reanalyzed separately for males and females. This allowed us to obtain more information about the intestinal bacteria associated with MCI than if we had only analyzed mixed-sex groups.

The intestinal bacterial taxa associated with MCI that were common between the ALDEx2 analyses of males and females were also observed as MCI-associated intestinal bacteria in the mixed-sex analysis (Figure 2). The characteristics of the intestinal bacteria are shown in Table 4. Interestingly, *Clostridium*_XVIII [25] and *Ruminococcus* 2 [26], which are associated with the degradation of mucin (which plays an important role in the intestinal barrier mechanism), were more abundant in the MCI groups, whereas *Roseburia*, a butyrate-producing bacterium associated with the protection of intestinal barrier function via flagellin [27,28], was less abundant in the MCI groups. This composition of intestinal microbiota may decrease intestinal barrier function and increase intestinal barrier permeability (i.e., leaky gut). *Roseburia* is less abundant in the intestinal microbiota of patients with neurodegenerative diseases that include cognitive decline as a symptom, such as Alzheimer’s disease [29,30], Parkinson’s disease [31], and amyotrophic lateral sclerosis [32]. Not only is *Roseburia* less abundant in Chinese patients with cognitive impairment, but *Ruminococcus* 2 is more abundant [33]. Increased intestinal barrier permeability has been identified as a mechanism underlying the onset and progression of MCI and dementia [5]. Regardless of sex in patients with MCI, increased intestinal barrier permeability may result in increased translocation of substances present in the intestine (including substances derived from intestinal bacteria, such as bacterial components and products) into the bloodstream.

*Erysipelatoclostridium* was a more abundant taxon common to both sexes and the mixed-sex MCI groups (Figure 2, Table 4). *Erysipelatoclostridium ramosum* (formerly named *Clostridium ramosum*) has been reported to promote serotonin (5-hydroxytryptamine) secretion in the intestine by enterochromaffin cells [34]. Overproduced serotonin in the intestine cannot cross the BBB, but has the potential to increase the permeability of the BBB [44,45,46]. Increased BBB permeability can lead to brain inflammation, which is associated with MCI.

*Erysipelatoclostridium ramosum* also produces IgA protease [35]. In human intestinal secretions, the IgA1 and IgA2 subclasses with different structures are present as IgA in approximately even proportions, and the IgA protease of *E*. *ramosum* can degrade both (IgA1 and IgA2m(1) allotypes) [47]. In particular, its ability to degrade IgA2, which is resistant to many bacterial proteases, is thought to affect the intestinal immune system. IgA is not only secreted into the intestinal lumen, but is also maintained in the mucus layer on intestinal mucosal epithelial cells, where it controls the contact and adhesion of intestinal bacteria to intestinal mucosal epithelial cells and prevents their entry into the circulatory system [48]. The degradation of IgA by IgA proteases may promote the contact, colonization, and invasion of *E*. *ramosum* but also various intestinal bacteria in intestinal mucosal epithelial cells, leading to dysregulation of the intestinal microbiota and increased inflammation of intestinal mucosal epithelial cells. A recent study also reported that *Paraprevotella* protects IgA via the degradation of intestinal trypsin and contributes to maintaining high levels of IgA in the intestine [49]. *Paraprevotella* was less abundant in the female MCI group, which may promote IgA degradation and contribute to dysregulation of the intestinal microbiota.

*Eggerthella* was a more abundant taxon in the MCI groups (Figure 2, Table 4). *Eggerthella lenta* is involved in metabolizing bile acids [37]. Bile acid oxidation by *E. lenta* is inhibited under high levels of molecular hydrogen (H_2_) [50,51]. Bile acid oxidation by *E*. *lenta* is inhibited by H_2_, the conversion of primary bile acids to secondary bile acids by other intestinal bacteria proceeds, leading to an increase in the hydrophobicity of the colonic bile acid pool. In contrast, at low levels of H_2_, bile acid oxidation by *E*. *lenta* would increase and the conversion of primary to secondary bile acids by other intestinal bacteria would be inhibited. This results in a change in the hydrophobic–hydrophilic balance of the bile acid pool in the colon. Hydrophobic bile acids are more toxic to bacteria than hydrophilic bile acids, and changes in the hydrophobic–hydrophilic balance of the bile acid pool in the colon may affect the regulation of the intestinal microbiota composition. In previous coculture experiments, bile acid oxidation by *E*. *lenta* has been reported to reduce bile acid toxicity and alleviate growth inhibition of *E*. *ramosum* [52]. Interestingly, *Roseburia*, *Megasphaera*, and *Victivallis*, which were less abundant in both sexes and the mixed-sex MCI groups, were associated with H_2_ production, suggesting that patients with MCI may produce less H_2_ in their intestinal tract (Table 4). These considerations suggest that a combination of low levels of H_2_ and high levels of *Eggerthella* may contribute to the formation of intestinal microbiota in patients with MCI. In addition, dysregulation of bile acid synthesis and metabolism may affect cognition [6], and *Eggerthella* may affect MCI through bile acid metabolism.

In the Japanese population, H_2_ produced in the intestine tends to be used for acetic acid production rather than methanogenesis [53]. If H_2_ production is low in patients with MCI, then acetic acid production may also be low. A decrease in acetic acid leads to an increase in intestinal pH, which may not prevent the growth of intestinal bacteria sensitive to low pH and may lead to dysregulation of the intestinal microbiota. Acetic acid has also been reported to differentially regulate IgA reactivity to commensal bacteria, and a decrease in acetic acid levels may affect this [54].

The H_2_ produced in the intestine acts as a selective scavenger of hydroxyl radicals (·OH) and may play an important role in suppressing chronic inflammation in the body [55]. H_2_, which is smaller in size, can diffuse in and out of the bloodstream and pass through selective barriers such as the BBB, allowing it to act at different sites in the body. Previously, H_2_ intake was reported to improve cognitive function in patients with MCI [56,57]. In this study, *Roseburia*, *Megasphaera*, and *Victivallis*, which are associated with H_2_ production, were less abundant in the MCI groups. Decreased H_2_ production in the intestines may lead to chronic inflammation, which contributes to MCI.

In addition to the less abundant taxa shown in Table 4 in the MCI groups, *Ruminococcus*, *Turicibacter*, *Catenibacterium*, *Dialister*, *Coprobacillus*, *Akkermansia*, *Lawsonibacter*, *Anaerotruncus*, and *Eisenbergiella* were observed in the male MCI group. However, *Agathobacter*, *Veillonella*, *Sutterella*, *Coprococcus*, *Bifidobacterium*, *Slackia*, *Anaerostipes*, *Leuconostoc*, *Paraprevotella*, *Ligilactobacillus*, *Holdemanella*, *Lactococcus*, *Clostridium*_XlVb, and *Megamonas* were observed in the female MCI group, in addition to those listed as less abundant in Table 4 in the MCI groups. Among these, the H_2_-producing capacity was reported for *Ruminococcus* [58] and *Agathobacter* [59], which had large absolute values of effect size in the male and female groups, respectively (Figure 2). These two taxa may also contribute to the reduced H_2_ production in males and females affected by MCI. In addition, *Agathobacter* is phylogenetically closely related to *Roseburia* [59,60] and may play a role similar to *Roseburia* in the intestine.

In this study, *Oscillibacter* and *Megasphaera*, both related to the production of valeric acid, were less abundant and common to both sexes and the mixed-sex MCI groups (Figure 2, Table 4). Valeric acid potentially contributes to histone deacetylase (HDAC) inhibitory effects [41]. Epigenetic dysregulation via HDAC isoforms has been reported to contribute to neuronal dysfunction and cognitive decline in Alzheimer’s disease and MCI [61]. In patients with MCI, decreased production of valeric acid, which inhibits the activity of HDAC isoforms, may lead to epigenetic dysregulation and inflammation associated with MCI.

Propionic acid and butyric acid are HDAC inhibitors [62]. Germ-free (GF) rats supplemented with these substances showed inhibited HDAC1 expression, reduced microglial activation, the release of pro-inflammatory cytokines and chemokines in the anterior cingulate cortex and hippocampus, and alleviated neuroinflammation [63]. Oral treatment of GF rats with *Roseburia hominis*, which produces propionic and butyric acids, alleviated neuroinflammation through a mechanism similar to that described above [63]. *Roseburia* was a common less abundant taxon in male and female MCI groups, and decreased production of propionic acid and butyric acid by intestinal bacteria such as *Roseburia* might also lead to increased neuroinflammation.

Butyric acid activates the secretion of brain-derived neurotrophic factors that play an important role in cognitive function [64]. This finding also suggests that a decrease in butyric acid-producing bacteria, such as *Roseburia*, may affect MCI.

*Flavonifractor*, a more abundant taxon common to both sexes and the mixed-sex MCI groups (Figure 2, Table 4), is involved in the conversion of catechins, a class of bioactive polyphenols abundant in the human diet [36]. However, it remains unclear whether catechin conversion itself is associated with MCI. Interestingly, *E*. *lenta* and *Flavonifractor plautii* have also been isolated from an epicatechin-converting human fecal suspension as bacteria involved in catechin conversion [36]. *Flavonifractor* and *Eggerthella* may have similar catalytic capacities, including catechin conversion. *Flavonifractor* may be involved in physiological actions similar to *Eggerthella* in the intestine.

*Prevotella* was a less abundant taxon common to both sexes and the mixed-sex MCI groups (Figure 2, Table 4). Since *Prevotella* correlates with plant-rich diets abundant in carbohydrates and fibers [39], the decrease in *Prevotella* might have been related to the diet of the MCI and control groups in this study. However, a previous study found *Prevotella* to be more abundant in the MCI group [8], unlike the results of this study. *Prevotella* is a genus with high genetic diversity, and some species may have potential roles as intestinal pathogens [39,65]. This may have led to discrepancies in the results.

In addition to the taxa listed in Table 4, *Rothia*, *Streptococcus*, *Intestinibacter*, *Bifidobacterium*, *Fournierella*, *Anaerostipes*, *Merdimonas*, *Parasutterella*, *Veillonella*, *Faecalicatena*, and *Adlercreutzia* were also more abundant in the male MCI group (Figure 2). This may be attributed to the absence of growth inhibition due to the aforementioned dysregulation of the intestinal microbiota. For example, *Rothia*, *Streptococcus*, and *Veillonella* are part of the normal oral microbiota [66,67] but also grow in the intestine. Meanwhile, in the female MCI group, *Ruthenibacterium*, *Enterocloster*, *Anaerotignum*, *Dysosmobacter*, *Turicibacter*, *Romboutsia*, *Eisenbergiella*, *Intestinimonas*, *Sellimonas*, *Bacteroides*, *Frisingicoccus*, *Hungatella*, *Gordonibacter*, *Neglecta*, *Negativibacillus*, *Fusobacterium*, *Massilimicrobiota*, *Parabacteroides*, *Clostridium*_XlVa, *Blautia*, *Merdimonas*, *Clostridium*_IV, *Coprobacillus*, *Anaerotruncus*, *Akkermansia*, *Ihubacter*, and *Anaeromassilibacillus* were more abundant (Figure 2), in addition to the taxa shown in Table 4. In contrast to males, oral-related taxa (*Rothia*, *Streptococcus*, and *Veillonella*) were not more abundant in females. The results indicate that many of the more abundant taxa in the MCI group differed by sex.

Furthermore, there were taxa in which conflicting associations with MCI were observed according to sex (*Eisenbergiella*, *Turicibacter*, *Akkermansia*, *Coprobacillus*, *Anaerotruncus*, *Anaerostipes*, *Bifidobacterium*, and *Veillonella*) (Figure 2). Although male and female patients with MCI may show some common dysregulation of the intestinal microbiota, the results of this study suggest that the taxa of intestinal bacteria that increase or decrease are significantly influenced by sex. However, it is unclear whether there are other factors besides sex differences in the intestinal microbiota that lead to sex-dependent bacterial increases or decreases in patients with MCI; further studies are needed to elucidate this.

Various substances such as lipopolysaccharides (LPS), lipoproteins, peptidoglycans (PGN), flagellins, and nucleic acids derived from intestinal bacterial cells have the potential to stimulate the host’s immune system and induce inflammation [68]. Thus, for all intestinal bacteria observed as more abundant in the MCI groups, if substances derived from them were to pass through the intestinal barrier, they could cause inflammation, leading to cognitive decline.

LPS, PGN, DNA, and bacterial amyloids from intestinal bacteria are associated with the induction of Alzheimer’s and Parkinson’s diseases [69]. In addition, trimethylamine-N-oxide, an intestinal microbiota-dependent metabolite, potentially exacerbates cognitive impairment in Alzheimer’s [69]. The location and degree of inflammation may differ depending on the type and number of substances permeating the intestine. This may lead to diverse disease states and changes in the name of the disease after its progression.

No significant differences in intestinal microbiota α-diversity were observed between the MCI and control groups (Table 1, Table 2 and Table 3). The β-diversity analysis of the mixed-sex group indicated that the composition of the intestinal microbiota differed between the MCI and control groups (Figure 1). In the sex-specific β-diversity reanalysis, significant differences were found between the MCI and control groups in females (Figure 3). Previous studies show no consistency in results for α-diversity and β-diversity [7,8,10,11,12,13]. This suggests that it is difficult to clearly distinguish between the MCI and control groups in terms of diversity.

This study showed that sex-specific risk estimation models for MCI based on intestinal microbiota were useful in distinguishing the MCI group from the control group (males: AUC = 0.75, females: AUC = 0.87) (Figure 5). In general, an AUC value of 0.5 suggests no discrimination, 0.7 to 0.8 is acceptable, 0.8 to 0.9 is excellent, and more than 0.9 is outstanding [70]. Intestinal microbiota testing using stool samples is non-invasive and can be easily performed. Incorporating these risk estimation models for MCI into intestinal microbiota testing using stool samples could lead to an efficient system for screening individuals with MCI or those at high risk of MCI.

Patients with MCI are also at risk of progressing to Alzheimer’s disease and other dementias. Prevalence tends to be higher in males with MCI, while it is higher in females with Alzheimer’s disease [71]. Because females have an advantage over males in verbal memory, conventional verbal memory tests may miss early signs of Alzheimer’s disease in females, possibly resulting in the underdiagnosis of MCI [72]. In contrast, MCI risk estimation based on intestinal microbiota is not affected by verbal memory and may solve this issue. In the future, the early detection of MCI by applying the results from this study is expected to lead to strategies that can delay or prevent the progression from MCI to dementia through intervention approaches, such as diet and supplements. Therefore, risk estimation of MCI in this study is an unprecedented approach to preventing dementia.

Sample size influences the statistical significance of the results [73]; therefore, the small sample size of this study is a limitation. This study focused on analyzing the effect size, which is independent of sample size, to identify the intestinal bacteria that differed between the MCI and control groups. Other limitations of this study include the age of the study participants (analysis was limited to participants in their 70s) and the lack of control for comorbidities as well as the educational history of patients with MCI. Therefore, studies with larger sample sizes controlling for various confounding factors should be conducted to test the hypotheses proposed in this study.

## 5. Conclusions

In this study, we identified the intestinal bacteria associated with MCI by comparing the intestinal microbiota of MCI and disease-free control groups of Japanese individuals in their 70s. In particular, we showed that it is possible to obtain information that cannot be obtained from mixed-sex analyses alone by performing an additional sex-specific reanalysis. Therefore, it is important to consider sex in studies investigating the association between MCI and intestinal microbiota.

**Figure 6:**
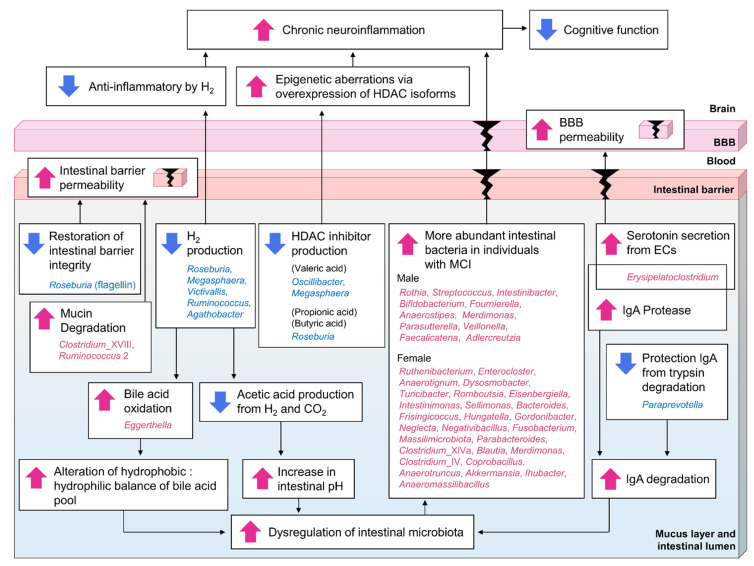
Hypothetical conceptual diagram of the influence of intestinal microbiota in individuals with MCI. Compared to the disease-free control group, the red-colored taxa were more abundant in the MCI group and the blue-colored taxa were less abundant. Red arrows indicate an increase; blue arrows indicate a decrease. Black arrows indicate the direction of the relationship. BBB: blood–brain barrier; ECs: enterochromaffin cells.

Information on the intestinal bacteria associated with MCI led to nine hypotheses (a summary of these hypotheses is shown in Figure 6):

1. A decrease in the abundance of H_2_-producing *Roseburia*, *Megasphaera*, *Victivallis*, *Ruminococcus*, and *Agathobacter*, and an increase in the abundance of *Eggerthella*, which oxidizes bile acids in an H_2_ concentration-dependent manner, leads to dysregulation of the intestinal microbiota.
2. A decrease in the abundance of H_2_-producing *Roseburia*, *Megasphaera*, *Victivallis*, *Ruminococcus*, and *Agathobacter* leads to dysregulation of the intestinal microbiota by indirectly causing an increase in intestinal pH due to decreased acetic acid production.
3. An increase in the abundance of IgA protease-producing *Erysipelatoclostridium* and a decrease in the abundance of *Paraprevotella*, which protects IgA via the degradation of intestinal trypsin, leads to dysregulation of the intestinal microbiota and increased inflammation of intestinal epithelial cells.
4. An increase in the abundance of *Clostridium*_XVIII and *Ruminococcus* 2, which are associated with mucin degradation, and a decrease in the abundance of *Roseburia*, associated with the protection of intestinal barrier function, leads to increased intestinal barrier permeability.
5. An increase in the abundance of *Erysipelatoclostridium*, which promotes serotonin secretion in the intestine, leads to increased BBB permeability.
6. The taxa of intestinal bacteria that are more abundant due to dysregulation of the intestinal microbiota differ between sexes, but they contribute to inflammation.
7. A decrease in the abundance of HDAC-inhibitor-producing bacteria (such as *Oscillibacter* and *Megasphaera*, which produce valeric acid, and *Roseburia*, which produces propionic acid and butyric acid) leads to epigenetic dysregulation due to excess HDAC activity, leading to increased inflammation.
8. A decrease in the abundance of H_2_-producing *Roseburia*, *Megasphaera*, *Victivallis*, *Ruminococcus*, and *Agathobacter* leads to decreased reactive oxygen species removal by H_2_ and increased chronic inflammation.
9. The composition of the intestinal microbiota in MCI-affected individuals leads to dysregulation of the intestinal microbiota, increased intestinal barrier and BBB permeability, and increased chronic neuroinflammation, which, when sustained over time, ultimately leads to cognitive decline.

Future hypothesis testing would lead to a better understanding of the mechanisms underlying the onset and progression of MCI and aid the development of methods for its treatment and prevention. Interestingly, the results of this study suggest that H_2_-producing bacteria play an important role in the regulation of intestinal microbiota. Dysregulation of the intestinal microbiota may be a mechanism of dysbiosis. A decrease in H_2_-producing bacteria in the intestine might trigger dysbiosis.

The risk estimation model for MCI based on the intestinal microbiota constructed in this study could discriminate between the MCI and control groups. Incorporating this into intestinal microbiota testing could provide an efficient system for screening individuals with MCI or those at high risk of MCI.

## Figures and Tables

**Table 1:** Characteristics and α-diversity indices of participants in the mild cognitive impairment (MCI) and control groups.

	MCI (*n* = 29)	Control (*n* = 40)	*p*-Value ^1^
Sex (male/female, *n*)	11/18	17/23	0.894
Age (years, mean ± SD)	74.2 ± 2.1	72.6 ± 2.7	<0.001
Body mass index (kg/m^2^, mean ± SD)	21.4 ± 2.5	21.2 ± 2.6 ^2^	0.775
α-diversity indices (mean ± SD):			
Shannon	2.66 ± 0.25	2.66 ± 0.29	0.976
Simpson	0.88 ± 0.03	0.88 ± 0.04	0.731
Richness	50.5 ± 15.2	46.1 ± 13.7	0.173
Pielou	0.69 ± 0.05	0.70 ± 0.06	0.122

^1^ The *p*-value for sex was determined by a chi-square test and the other factors were determined by the Wilcoxon rank-sum test; ^2^ Calculated excluding one participant with no data.

**Table 2:** Age, body mass index, and α-diversity indices of male MCI and control groups.

	MCI (*n* = 11)	Control (*n* = 17)	*p*-Value ^1^
Age (years, mean ± SD)	73.2 ± 1.0	71.7 ± 2.1	0.001
Body mass index (kg/m^2^, mean ± SD)	21.5 ± 2.8	21.6 ± 2.9	0.689
α-diversity indices (mean ± SD):			
Shannon	2.57 ± 0.25	2.76 ± 0.33	0.100
Simpson	0.88 ± 0.03	0.89 ± 0.04	0.175
Richness	46.9 ± 17.1	48.4 ± 15.9	0.851
Pielou	0.69 ± 0.06	0.72 ± 0.06	0.122

^1^ Wilcoxon rank-sum test.

**Table 3:** Age, body mass index, and α-diversity indices of female MCI and control groups.

	MCI (*n* = 18)	Control (*n* = 23)	*p*-Value ^1^
Age (years, mean ± SD)	74.9 ± 2.3	73.3 ± 2.9	0.052
Body mass index (kg/m^2^, mean ± SD)	21.4 ± 2.4	20.9 ± 2.3 ^2^	0.438
α-diversity indices (mean ± SD):			
Shannon	2.71 ± 0.23	2.58 ± 0.22	0.113
Simpson	0.88 ± 0.03	0.87 ± 0.03	0.383
Richness	52.7 ± 13.5	44.5 ± 11.7	0.092
Pielou	0.69 ± 0.04	0.69 ± 0.05	0.785

^1^ Wilcoxon rank-sum test; ^2^ Calculated excluding one participant with no data.

**Table 4:** Intestinal bacteria associated with MCI common to both sexes and mixed-sex groups and their known characteristics.

Taxa	Known Characteristics of Members of the Taxon	Reference
More abundant taxa in the MCI groups:		
*Clostridium*_XVIII	*Clostridium cocleatum* is involved in mucin degradation.	[25]
*Erysipelatoclostridium*	*Erysipelatoclostridium ramosum* promotes intestinal serotonin secretion, therefore promoting the development of intestinal lipid absorption and obesity.	[34]
	*Erysipelatoclostridium ramosum* produces IgA proteases that help evade host immune defenses.	[35]
*Ruminococcus* 2	*Ruminococcus torques* is involved in the degradation of mucin.	[26]
*Flavonifractor*	*Flavonifractor plautii* is involved in the degradation of catechins.	[36]
*Eggerthella*	*Eggerthella lenta* is involved in the inactivation of the cardiac drug digoxin, various reactions of dietary phytochemicals, dehydroxylation of catechols, and metabolism of bile acids.	[37]
Less abundant taxa in the MCI groups:		
*Roseburia*	*Roseburia* species produce butyric acid.	[27]
	Flagellin from *Roseburia intestinalis* is involved in protecting the intestinal barrier function.	[28]
	*Roseburia cecicola* produces H_2_.	[38]
*Prevotella*	*Prevotella* correlates with plant-rich diets, abundant in carbohydrates and fibers.	[39]
*Oscillibacter*	*Oscillibacter valericigenes* produces valeric acid.	[40]
*Megasphaera*	*Megasphaera massiliensis* produces valeric acid.	[41]
	*Megasphaera elsdenii* produces H_2_.	[42]
*Victivallis*	*Victivallis vadensis* produces H_2_.	[43]

## Data Availability

The data presented in this study are available upon request from the corresponding author. These data are not publicly available because of privacy concerns.

## Supplementary Materials

The following supporting information can be downloaded at: https://www.mdpi.com/article/10.3390/biomedicines11071789/s1, Figure S1: Constructed structural equation model for males and its values for each parameter; Table S1: Existing diseases in patients with MCI; Table S2: The male structural equation modeling output as shown in Figure 4a; Table S3: The female structural equation modeling output as shown in Figure 4b; and Table S4: The male structural equation modeling output as shown in Supplementary Figure S1.
