# Supplementary material for: Characteristics of Intestinal Microbiota in Japanese Patients with Mild Cognitive Impairment and a Risk-Estimating Method for the Disorder
Source: Biomedicines. 2023 Jun 22;11(7):1789. doi: 10.3390/biomedicines11071789 (PMC10376419; doi:10.3390/biomedicines11071789)
Supplement: Supplementary file 1 [file biomedicines-11-01789-s001.zip › biomedicines-2456205-SI.pdf]

biomedicines

Characteristics of Intestinal Microbiota in Japanese Patients with Mild Cognitive Impairment and a Risk-Estimating Method for the Disorder

Kouta Hatayama [1,*], Aya Ebara [1], Kana Okuma [1], Hidetaka Tokuno [1], Kazumi Hasuko [1], Hiroaki Masuyama [1], Iyoko Ashikari [2], and Takuji Shirasawa [3]

[1] Symbiosis Solutions Inc., Tokyo 101-0064, Japan
[2] Ashikari Clinic, Tokyo 164-0011, Japan
[3] Ochanomizu Health and Longevity Clinic, Tokyo 101-0062, Japan
* Correspondence: hatayama@symbiosis-solutions.co.jp; Tel.: +81-3-6275-0878

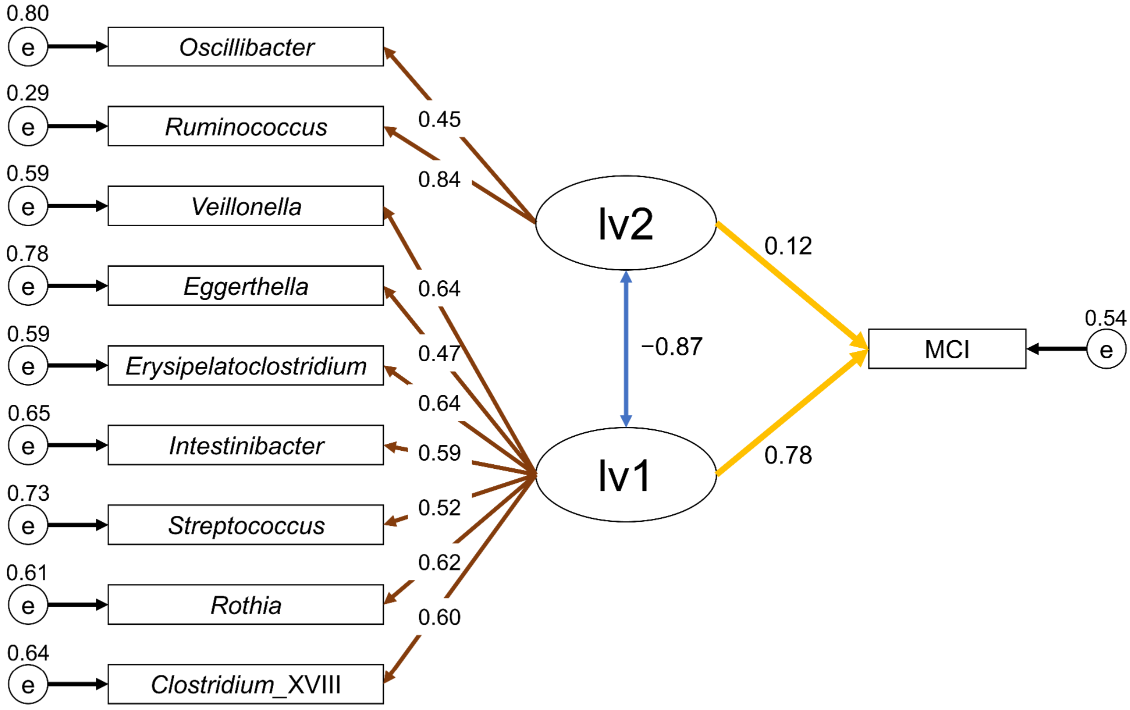

Supplementary Figure S1. Constructed structural equation model for males and its values for each parameter. Ellipse (lv1 or lv2), rectangle (MCI or taxa name) and circle (e) represent latent variables, observed variables, and residual terms, respectively. The values in the small circles, double-headed blue arrow, brown arrow, and yellow arrow represent residual variance, correlation coefficient, loading value, and path coefficient, respectively. The goodness-of-fit indices of the SEM were goodness-of-fit index (GFI) = 0.94, adjusted GFI (AGFI) = 0.88, and the root mean square error of approximation (RMSEA) = < 0.01. The detailed results are shown in Supplementary Table S4.

Supplementary Table S1. Exiting diseases in patients with MCI.

Disease	Male (*n*)	Female (*n*)
Arrhythmia	1	0
Arthralgia	0	1
Benign prostatic hyperplasia	1	0
Cataract	2	2
Cerebrovascular disease	1	0
Chronic gastritis	0	1
Chronic kidney disease	2	0
Constipation	1	4
Diseases of circulatory system	1	0
Dyslipidemia	2	2
Gout	1	0
Hashimoto's thyroiditis	1	1
Hypertension	4	4
Hypothyroidism	0	1
liver cirrhosis	1	0
Low back pain	1	1
Malaise and fatigue	0	1
Multiple myeloma	0	1
Osteoporosis	0	4
Overactive bladder	0	1
Periodontal disease	1	0
Pollinosis	1	1
Reflux esophagitis	1	0
Sensitivity to cold	0	2
Sleep disorder	0	1
Type 1 diabetes	0	1
Underweight	0	1

Supplementary Table S2. The male structural equation modeling output as shown in Figure 4a. NA, not available.

**lhs**[a]	**op**[b]	**rhs**[c]	**est.std**[d]	**se**[e]	**z**[f]	***p*-value**[g]	**ci.lower**[h]	**ci.upper**[i]
lv1	=~	*Clostridium*_XVIII	0.57	0.19	2.98	<0.01	0.19	0.94
lv1	=~	*Rothia*	0.66	0.16	4.02	<0.01	0.34	0.98
lv1	=~	*Streptococcus*	0.57	0.18	3.23	<0.01	0.22	0.92
lv1	=~	*Intestinibacter*	0.59	0.20	3.02	<0.01	0.21	0.97
lv1	=~	*Erysipelatoclostridium*	0.61	0.20	3.11	<0.01	0.23	1.00
lv1	=~	*Eggerthella*	0.49	0.17	2.82	<0.01	0.15	0.83
lv1	=~	*Veillonella*	0.56	0.19	3.03	<0.01	0.20	0.93
MCI	~	lv1	0.69	0.20	3.45	<0.01	0.30	1.08
*Clostridium*_XVIII	~~	*Clostridium*_XVIII	0.68	0.21	3.18	<0.01	0.26	1.10
Rothia	~~	*Rothia*	0.57	0.21	2.65	0.01	0.15	0.99
Streptococcus	~~	*Streptococcus*	0.67	0.20	3.34	<0.01	0.28	1.07
Intestinibacter	~~	*Intestinibacter*	0.65	0.23	2.82	<0.01	0.20	1.10
Erysipelatoclostridium	~~	*Erysipelatoclostridium*	0.62	0.24	2.56	0.01	0.15	1.10
Eggerthella	~~	*Eggerthella*	0.76	0.17	4.47	<0.01	0.43	1.09
Veillonella	~~	*Veillonella*	0.68	0.21	3.28	<0.01	0.28	1.09
MCI	~~	MCI	0.52	0.28	1.88	0.06	−0.02	1.07
lv1	~~	lv1	1.00	0	NA	NA	1	1
*Clostridium*_XVIII	~1		<0.01	0.21	<0.01	1.00	−0.41	0.41
Rothia	~1		<0.01	0.23	<0.01	1.00	−0.45	0.45
Streptococcus	~1		<0.01	0.21	<0.01	1.00	−0.41	0.41
Intestinibacter	~1		<0.01	0.20	<0.01	1.00	−0.38	0.38
Erysipelatoclostridium	~1		<0.01	0.22	<0.01	1.00	−0.43	0.43
Eggerthella	~1		<0.01	0.20	<0.01	1.00	−0.40	0.40
Veillonella	~1		0	0.27	0	1.00	−0.52	0.52
MCI	~1		0	0	NA	NA	0	0
lv1	~1		0	0	NA	NA	0	0

[a] Variable name that appears on the left-hand side of the formula
[b] Operator indicates the following formula types: =~, latent variable definition; ~, regression; ~~, (residual) (co)variance; and ~1, intercept
[c] Variable name that appears on the right-hand side of the formula
[d] Standardized parameter estimates
[e] Standard error for each estimated parameter
[f] Z-value
[g] *p*-value for testing the null hypothesis that the parameter value equals zero in the population
[h] Lower 95% confidence intervals for standardized parameters
[i] Upper 95% confidence intervals for standardized parameters

Supplementary Table S3. The female structural equation modeling output as shown in Figure 4b. NA, not available.

lhs[a]	op[b]	rhs[c]	est.std[d]	se[e]	z[f]	*p*-value[g]	ci.lower[h]	ci.upper[i]
lv1	=~	*Ruthenibacterium*	0.65	0.13	4.92	<0.01	0.39	0.90
lv1	=~	*Enterocloster*	0.80	0.09	9.34	<0.01	0.63	0.96
lv1	=~	*Erysipelatoclostridium*	0.71	0.11	6.67	<0.01	0.50	0.92
lv1	=~	*Flavonifractor*	0.77	0.11	6.83	<0.01	0.55	0.99
lv1	=~	*Dysosmobacter*	0.62	0.14	4.53	<0.01	0.35	0.88
lv1	=~	*Eggerthella*	0.74	0.14	5.42	<0.01	0.47	1.00
lv1	=~	*Sellimonas*	0.58	0.15	4.00	<0.01	0.30	0.87
lv1	=~	*Bacteroides*	0.73	0.10	7.59	<0.01	0.54	0.91
lv2	=~	*Veillonella*	0.57	0.18	3.12	<0.01	0.21	0.94
lv2	=~	*Ligilactobacillus*	0.48	0.25	1.96	0.05	<0.01	0.96
lv2	=~	*Megamonas*	0.85	0.25	3.40	<0.01	0.36	1.34
MCI	~	lv1	0.52	0.18	2.94	<0.01	0.17	0.87
MCI	~	lv2	−0.52	0.22	−2.36	0.02	−0.95	−0.09
Ruthenibacterium	~~	*Ruthenibacterium*	0.58	0.17	3.43	<0.01	0.25	0.92
Enterocloster	~~	*Enterocloster*	0.36	0.14	2.68	0.01	0.10	0.63
Erysipelatoclostridium	~~	*Erysipelatoclostridium*	0.49	0.15	3.20	<0.01	0.19	0.79
Flavonifractor	~~	*Flavonifractor*	0.41	0.17	2.33	0.02	0.06	0.75
Dysosmobacter	~~	*Dysosmobacter*	0.62	0.17	3.69	<0.01	0.29	0.95
Eggerthella	~~	*Eggerthella*	0.46	0.20	2.31	0.02	0.07	0.85
Sellimonas	~~	*Sellimonas*	0.66	0.17	3.89	<0.01	0.33	0.99
Bacteroides	~~	*Bacteroides*	0.47	0.14	3.38	<0.01	0.20	0.74
Veillonella	~~	*Veillonella*	0.67	0.21	3.16	<0.01	0.25	1.08
Ligilactobacillus	~~	*Ligilactobacillus*	0.77	0.24	3.26	<0.01	0.31	1.23
Megamonas	~~	*Megamonas*	0.28	0.42	0.67	0.51	−0.55	1.11
MCI	~~	MCI	0.24	0.20	1.15	0.25	−0.17	0.64
lv1	~~	lv1	1.00	0	NA	NA	1.00	1.00
lv2	~~	lv2	1.00	0	NA	NA	1.00	1.00
lv1	~~	lv2	−0.41	0.17	−2.40	0.02	−0.75	−0.08
Ruthenibacterium	~1		<0.01	0.17	<0.01	1.00	−0.33	0.33
Enterocloster	~1		<0.01	0.16	<0.01	1.00	−0.31	0.31
Erysipelatoclostridium	~1		<0.01	0.16	<0.01	1.00	−0.31	0.31
Flavonifractor	~1		<0.01	0.16	<0.01	1.00	−0.31	0.31
Dysosmobacter	~1		<0.01	0.17	<0.01	1.00	−0.34	0.34
Eggerthella	~1		<0.01	0.17	<0.01	1.00	−0.33	0.33
Sellimonas	~1		<0.01	0.24	<0.01	1.00	−0.47	0.47
Bacteroides	~1		<0.01	0.19	<0.01	1.00	−0.38	0.38
Veillonella	~1		<0.01	0.17	<0.01	1.00	−0.34	0.34
Ligilactobacillus	~1		<0.01	0.32	<0.01	1.00	−0.64	0.64
Megamonas	~1		<0.01	0.52	<0.01	1.00	−1.02	1.02
MCI	~1		0	0	NA	NA	0	0
lv1	~1		0	0	NA	NA	0	0
lv2	~1		0	0	NA	NA	0	0

[a] Variable name that appears on the left-hand side of the formula

[b] Operator indicates the following formula types: =~, latent variable definition; ~, regression; ~~, (residual) (co)variance; and ~1, intercept

[c] Variable name that appears on the right-hand side of the formula

[d] Standardized parameter estimates

[e] Standard error for each estimated parameter

[f] Z-value
[g] *p*-value for testing the null hypothesis that the parameter value equals zero in the population
[h] Lower 95% confidence intervals for standardized parameters
[i] Upper 95% confidence intervals for standardized parameters

Supplementary Table S4. The male structural equation modeling output as shown in Supplementary Figure S1. NA, not available.

lhs[a]	op[b]	rhs[c]	est.std[d]	se[e]	z[f]	*p*-value[g]	ci.lower[h]	ci.upper[i]
lv1	=~	*Clostridium*_XVIII	0.60	0.18	3.35	<0.01	0.25	0.95
lv1	=~	*Rothia*	0.62	0.16	3.84	<0.01	0.31	0.94
lv1	=~	*Streptococcus*	0.52	0.19	2.76	0.01	0.15	0.88
lv1	=~	*Intestinibacter*	0.59	0.19	3.17	<0.01	0.23	0.96
lv1	=~	*Erysipelatoclostridium*	0.64	0.20	3.24	<0.01	0.25	1.02
lv1	=~	*Eggerthella*	0.47	0.17	2.77	0.01	0.14	0.81
lv1	=~	*Veillonella*	0.64	0.18	3.64	<0.01	0.29	0.98
lv2	=~	*Ruminococcus*	0.84	0.33	2.58	0.01	0.20	1.48
lv2	=~	*Oscillibacter*	0.45	0.18	2.54	0.01	0.10	0.79
MCI	~	lv1	0.78	1.05	0.74	0.46	−1.28	2.83
MCI	~	lv2	0.12	1.09	0.11	0.91	−2.02	2.26
*Clostridium*_XVIII	~~	*Clostridium*_XVIII	0.64	0.22	2.97	<0.01	0.22	1.06
Rothia	~~	*Rothia*	0.61	0.20	3.00	<0.01	0.21	1.01
Streptococcus	~~	*Streptococcus*	0.73	0.19	3.81	<0.01	0.36	1.11
Intestinibacter	~~	*Intestinibacter*	0.65	0.22	2.93	<0.01	0.21	1.08
Erysipelatoclostridium	~~	*Erysipelatoclostridium*	0.59	0.25	2.36	0.02	0.10	1.09
Eggerthella	~~	*Eggerthella*	0.78	0.16	4.81	<0.01	0.46	1.09
Veillonella	~~	*Veillonella*	0.59	0.22	2.65	0.01	0.15	1.03
Ruminococcus	~~	*Ruminococcus*	0.29	0.55	0.54	0.59	−0.78	1.37
Oscillibacter	~~	*Oscillibacter*	0.80	0.16	5.13	<0.01	0.50	1.11
MCI	~~	MCI	0.54	0.30	1.81	0.07	−0.04	1.13
lv1	~~	lv1	1.00	0	NA	NA	1.00	1.00
lv2	~~	lv2	1.00	0	NA	NA	1.00	1.00
lv1	~~	lv2	−0.87	0.16	−5.44	<0.01	−1.18	−0.56
*Clostridium*_XVIII	~1		<0.01	0.21	<0.01	1.00	−0.41	0.41
Rothia	~1		<0.01	0.23	<0.01	1.00	−0.45	0.45
Streptococcus	~1		<0.01	0.21	<0.01	1.00	−0.41	0.41
Intestinibacter	~1		<0.01	0.20	<0.01	1.00	−0.38	0.38
Erysipelatoclostridium	~1		<0.01	0.22	<0.01	1.00	−0.43	0.43
Eggerthella	~1		<0.01	0.20	<0.01	1.00	−0.40	0.40
Veillonella	~1		0	0.27	0	1.00	−0.52	0.52
Ruminococcus	~1		<0.01	0.21	<0.01	1.00	−0.41	0.41
Oscillibacter	~1		0	0.21	0	1.00	−0.41	0.41
MCI	~1		0	0	NA	NA	0	0
lv1	~1		0	0	NA	NA	0	0
lv2	~1		0	0	NA	NA	0	0

[a] Variable name that appears on the left-hand side of the formula
[b] Operator indicates the following formula types: =~, latent variable definition; ~, regression; ~~, (residual) (co)variance; and ~1, intercept
[c] Variable name that appears on the right-hand side of the formula
[d] Standardized parameter estimates
[e] Standard error for each estimated parameter
[f] Z-value
[g] *p*-value for testing the null hypothesis that the parameter value equals zero in the population
[h] Lower 95% confidence intervals for standardized parameters
[i] Upper 95% confidence intervals for standardized parameters
